# Human COQ9 Rescues a *coq9* Yeast Mutant by Enhancing Coenzyme Q Biosynthesis from 4-Hydroxybenzoic Acid and Stabilizing the CoQ-Synthome

**DOI:** 10.3389/fphys.2017.00463

**Published:** 2017-07-07

**Authors:** Cuiwen H. He, Dylan S. Black, Christopher M. Allan, Brigitte Meunier, Shamima Rahman, Catherine F. Clarke

**Affiliations:** ^1^Department of Chemistry and Biochemistry and the Molecular Biology Institute, University of California, Los AngelesLos Angeles, CA, United States; ^2^Institut de Biologie Intégrative de la Cellule, CEA, Centre National de la Recherche Scientifique, UPSud, Paris-Saclay UniversityGif-sur-Yvette, France; ^3^Metabolic Unit, Great Ormond Street Hospital for Children NHS Foundation TrustLondon, United Kingdom; ^4^Mitochondrial Research Group, Genetics and Genomic Medicine, UCL Great Ormond Street Institute of Child HealthLondon, United Kingdom

**Keywords:** human homolog, temperature-sensitive mutant, coenzyme Q, immunoprecipitation, *Saccharomyces cerevisiae*, mitochondrial metabolism

## Abstract

Coq9 is required for the stability of a mitochondrial multi-subunit complex, termed the CoQ-synthome, and the deamination step of Q intermediates that derive from para-aminobenzoic acid (pABA) in yeast. In human, mutations in the *COQ9* gene cause neonatal-onset primary Q_10_ deficiency. In this study, we determined whether expression of human *COQ9* could complement yeast *coq9* point or null mutants. We found that expression of human *COQ9* rescues the growth of the temperature-sensitive yeast mutant, *coq9-ts19*, on a non-fermentable carbon source and increases the content of Q_6_, by enhancing Q biosynthesis from 4-hydroxybenzoic acid (4HB). To study the mechanism for the rescue by human COQ9, we determined the steady-state levels of yeast Coq polypeptides in the mitochondria of the temperature-sensitive yeast *coq9* mutant expressing human *COQ9*. We show that the expression of human *COQ9* significantly increased steady-state levels of yeast Coq4, Coq6, Coq7, and Coq9 at permissive temperature. Human COQ9 polypeptide levels persisted at non-permissive temperature. A small amount of the human COQ9 co-purified with tagged Coq6, Coq6-CNAP, indicating that human COQ9 interacts with the yeast Q-biosynthetic complex. These findings suggest that human COQ9 rescues the yeast *coq9* temperature-sensitive mutant by stabilizing the CoQ-synthome and increasing Q biosynthesis from 4HB. This finding provides a powerful approach to studying the function of human COQ9 using yeast as a model.

## Introduction

Coenzyme Q (Q) is a lipid that functions as an electron and proton carrier in the mitochondrial respiratory chain and as a lipid-soluble antioxidant (Turunen et al., 2004). Q is composed of a redox-active benzoquinone ring and a polyisoprenoid side chain, which contains six isoprene units in *Saccharomyces cerevisiae* (Q_6_) and ten isoprene units in humans (Q_10_) (Crane and Barr, 1985). Both yeast and human cells are able to use 4-hydroxybenzoic acid (4HB), resveratrol, and coumarate to synthesize Q (Xie et al., 2015). However, while para-aminobenzoic acid (pABA) is a ring precursor for Q in yeast (Marbois et al., 2010), mammalian cells cannot synthesize Q from pABA (Xie et al., 2015). The biosynthetic pathway of Q is highly conserved among species. Genes involved in Q biosynthesis in yeast include *COQ1-10, YAH1*, and *ARH1*, all of which have human homologs (Desbats et al., 2015). A new gene involved in yeast Q biosynthesis was recently identified as *COQ11* (Allan et al., 2015), and whether the human homolog is also involved in Q biosynthesis requires further investigation. Most of the human homologs of yeast *COQ* genes rescue the corresponding yeast *coq* mutants. For example, co-expression of *PDSS1* and *PDSS2* rescue the yeast *coq1* mutant (Kawamukai, 2015) and expression of *COQ8A* (formerly identified as *ADCK3*) rescues the yeast *coq8* mutant (Xie et al., 2011). Similarly expression of human *COQ2, COQ3, COQ4, COQ6, COQ7, COQ10A*, (Wang and Hekimi, 2013; Desbats et al., 2015), or *COQ5* (Nguyen et al., 2014) each rescue the corresponding yeast *coq* mutant.

Yeast Coq9 is required for the function of Coq6 and Coq7 polypeptides in Q_6_ biosynthesis, and is essential for the stability of the CoQ synthome (Hsieh et al., 2007; He et al., 2014). Yeast Coq9 function is not fully understood, but it is required for the deamination of nitrogen-containing Q intermediates derived from pABA. For example, imino-demethoxy-Q_6_ (IDMQ_6_) and 3-hexaprenyl-4-aminophenol (4-AP) accumulate in the yeast *coq9* null mutant fed pABA in the setting of *COQ8* over-expression (Xie et al., 2012). The over-expression of yeast *COQ8* in several of the *coq* null mutants has been shown to stabilize the Q-biosynthetic complex and to facilitate formation of late-stage Q-intermediates (Xie et al., 2012). A distinct nitrogen-containing Q-intermediate, imino-demethyl-demethoxy Q_6_ (IDDMQ_6_) also accumulates in the pABA-fed yeast double *coq9* null + *coq5-5* point mutant (He et al., 2015). In contrast, the *coq5-5* single mutant is able to produce demethyl-demethoxy Q_6_ (DDMQ_6_) when fed pABA (Nguyen et al., 2014; He et al., 2015). These results suggest that yeast Coq9 functions either directly or indirectly to remove amino substituents from Q-intermediates that derive from pABA.

In previous work we generated a *coq9* temperature sensitive mutant plasmid using site-directed mutagenesis of the wild-type yeast *COQ9, coq9-ts19* (TS19). TS19 contains the following point mutations: Adenine-12
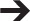
Guanine (a-12g), Adenine-93
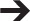
Guanine (a-93g), Glu55
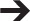
Gly (a164g; E55G), Arg107
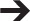
Gly (a319g; R107G), and Gln256
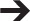
Leu (a767t; Q256L). This mutant grew as well as wild type at the permissive temperature of 25°C and grew poorly at the non-permissive temperature of 37°C. We found that at the non-permissive temperature, the levels of Coq9-ts19 polypeptide increased, but other yeast Coq polypeptides, Coq4, Coq5, Coq6, and Coq7 decreased and nitrogen-containing intermediates derived from pABA accumulated (He et al., 2015). This phenomenon is not understood; it may be that while the function of the Coq9-ts19 polypeptide is impaired with respect to wild-type Coq9, the mutations confer some form of thermal stability to the protein such that it is resistant to degradation at higher temperatures. Alternatively, the coq9-ts19 mutant contains two mutations upstream of the start codon, which may lead to more robust expression of the protein at the non-permissive temperature. In either case, the Coq9-ts19 polypeptide at the restrictive temperature is not fully functional, and leads to a drastic decrease in Q content (He et al., 2015). This in turn would impact the stability of the CoQ synthome and steady state levels of the Coq4, Coq5, and Coq7 polypeptides.

Taken together, these results indicate that yeast Coq9 is required to remove the nitrogen group of Q intermediates derived from pABA. Interestingly, human cells cannot synthesize Q from pABA (Xie et al., 2015). Thus, human COQ9 may lack the ability to aid in the deamination step(s) that are normally required in yeast Q biosynthesis from pABA.

It is clear that human COQ9 is required for Q_10_ biosynthesis; a mutation in the *COQ9* gene was identified in a patient with neonatal-onset primary Q_10_ deficiency (Duncan et al., 2009). The patient harbored a homozygous nonsense mutation in *COQ9*, resulting in a truncated COQ9 polypeptide (Arg244Ter), presented with neonatal lactic acidosis, and later developed multisystem disease including intractable seizures, global developmental delay, hypertrophic cardiomyopathy, and renal tubular dysfunction. Cultured skin fibroblasts from the patient were examined and found to contain low levels of Q_10_ relative to control subjects and a compound slightly more polar than Q_10_, suggestive of a Q_10_-intermediate (Duncan et al., 2009). Recently another patient who manifested fatal neonatal lactic acidosis and encephalopathy was diagnosed with a *COQ9* deficiency due to a splice site mutation that caused skipping of exons four and five (Danhauser et al., 2016). The content of Q_10_ in cultured fibroblasts derived from the patient was only 8–16% of normal levels, and demethoxy-Q_10_, a biosynthetic Q-intermediate, was readily detected (Danhauser et al., 2016). A third patient with a homozygous missense mutation in *COQ9* resulted in a missense COQ9 polypeptide (His62Arg), and was diagnosed with a primary deficiency in Q_10_ (Alfadhel et al., 2016).

Garcia-Corzo and co-workers generated a *Coq9* knockin mouse model by introducing the *Coq9-R239X* mutation that recapitulates the Arg244Ter human *COQ9* mutation (Garcia-Corzo et al., 2013). The *Coq9*^*R239X*/*R239X*^ homozygous knockin mice showed histologic and behavioral signs that mirrored mitochondrial encephalomyopathy associated with primary Q deficiency in human patients. A widespread Q deficiency was noted in these mice along with a dramatic reduction in the steady state level of the COQ7 polypeptide and accumulation of demethoxy-Q_9_ (DMQ_9_) (Garcia-Corzo et al., 2013). A second *Coq9* mouse model (*Coq9Q95X*) also demonstrated that COQ9 is required for Q biosynthesis (Luna-Sanchez et al., 2015). However, the Q deficiency in the *Coq9*^*Q95X*/*Q95X*^ mice is less severe than in the *Coq9*^*R239X*/*R239X*^ mice, probably due to differences in nonsense-mediated decay efficiency and in the stability of the CoQ synthome (Luna-Sanchez et al., 2015).

A recent study determined the structure of human COQ9 (Lohman et al., 2014). Human COQ9 functions as a dimer and has a hydrophobic interface that binds lipids and a surface patch that binds human COQ7 (Lohman et al., 2014). Taken together these results suggest that the COQ9 polypeptide is required for COQ7 function in yeast, mouse, and human Q biosynthesis.

Yeast has been a great model for the study of Q biosynthesis; it can be a powerful system to study human COQ proteins with unknown functions. In most cases, expression of human COQ homologs, rescue the corresponding yeast *coq* mutants (Wang and Hekimi, 2013; Hayashi et al., 2014). For example, human COQ6 expressed from a plasmid with yeast mitochondrial leader sequence rescued the yeast *coq6* null mutant for growth on a non-fermentable carbon source (Heeringa et al., 2011); in the yeast *coq5* null mutant over-expressing *COQ8*, expression of human COQ5 with its first 55 amino acids replaced by the first 54 amino acids of yeast Coq5 restored Q_6_ content and growth on a non-fermentable carbon source (Nguyen et al., 2014). However, expression of human COQ9 in yeast fails to restore Q biosynthesis in yeast *coq9* mutants (Duncan et al., 2009; Hayashi et al., 2014). In this study, we tested whether human COQ9 could rescue Q biosynthesis in distinct yeast *coq9* mutants. The results presented indicate that under certain conditions human COQ9 functions to restore yeast Q biosynthesis, but that the potential of yeast Coq9 to remove amino/imino groups from Q-intermediates is a functional role that is not shared with human COQ9.

## Materials and methods

### Yeast strains and growth media

*Saccharomyces cerevisiae* strains used in this study are listed in Table 1. Growth media used in this study included: YPD (2% glucose, 1% yeast extract, 2% peptone), YPGal (1% yeast extract, 2% peptone, 2% galactose, 0.1% dextrose), and YPG (3% glycerol, 1% yeast extract, 2% peptone), and were prepared as described (Burke et al, 2000). Synthetic Dextrose (SD)/Minimal medium consisted of 0.18% yeast nitrogen base without amino acids, 2% dextrose, 0.14% NaH_2_PO_4_, 0.5% (NH_4_)_2_SO_4_, and amino acids added to final concentrations as described (Barkovich et al., 1997). Selective SD/Minimal medium lacking uracil (SD–Ura) and selective SD/Minimal medium lacking uracil and leucine (SD–Ura–Leu) were similarly prepared. Agar plate media were prepared as described above and included 2% Bacto agar (Fisher).

**Table 1:** Genotype and Source of Yeast Strains.

**Strain**	**Genotype**	**Source**
W3031B	MAT α *ade2-1 his3-1,15 leu2-3,112 trp1-1 ura3-1*	R. Rothstein^a^
W303ΔCOQ4	MAT α *ade2-1 his3-1,15 leu2-3,112 trp1-1 ura3-1 coq4::TRP1*	Hsu et al., 2000
W303ΔCOQ6	MAT α *ade2-1 his3-1,15 leu2-3,112 trp1-1 ura3-1 coq6::LEU2*	Gin et al., 2003
W303ΔCOQ7	MAT α *ade2-1 his3-1,15 leu2-3,112 trp1-1 ura3-1 coq7::LEU2*	Marbois and Clarke, 1996
BY4741ΔCOQ9	MAT α *coq9Δ::kanMX4 his3Δ1 leu2Δ0 lys2Δ0 ura3Δ0*	Winzeler et al., 1999^b^
W303Δcoq9K	MAT α *ade2-1 his3-1,15 leu2-3,112 trp1-1 ura3-1 coq9::KanMX4*	This study
Coq6-CNAP	Mat α *ade2-1 his3-1,15 leu2-3,112 trp1-1 ura3-1 COQ6*::*COQ6*-*CNAP*-*HIS3*	Allan et al., 2015

a *Dr. Rodney Rothstein, Department of Human Genetics, Columbia University*.

b *European S. cerevisiae Archive for Functional Analysis (EUROSCARF), available on-line*.

### Construction of plasmids

*COQ8* was over-expressed in yeast with the p4HN4 plasmid (mc*COQ8*). The *COQ8* gene was cloned into pRS426, a multi-copy yeast shuttle vector, resulting in mc*COQ8* (Hsieh et al., 2004). To construct plasmids expressing human *COQ9*, we cloned human *COQ9* into pQM (Hsu et al., 1996) and pRCM (Allan et al., 2013). These are respectively low- and multi-copy vectors that express ORFs fused to the yeast Coq3 amino terminal mitochondrial leader sequence (amino acids 1–34) under control of the yeast *CYC1* promoter. Human *COQ9* was amplified from pBGcoq9, which contains the human *COQ9* ORF in YEpJB1-21-10 and is expressed from a constitutive PGK promoter (Duncan et al., 2009). The human *COQ9* ORF was amplified with Taq polymerase and primers Hcoq9F (5′-ATCGATATGGCGGCGG CGGCGGTAT-3′ with a ClaI restriction site at the 5′ end) and HcoqR (5′-GGTACCTC ACCGACGCTGGTTTAGACCTGTCAAGTTCTTGAGC-3′ with a KpnI restriction site at the 5′ end). PCR products were inserted into the TOPO vector resulting in a plasmid named HCOQ9TOPO. HCOQ9TOPO was digested with the restriction enzymes ClaI and KpnI (New England BioLabs) and inserted in pQM or pRCM prepared with ClaI and KpnI, resulting in the plasmids scHCOQ9 and mcHCOQ9, respectively. The nucleotide sequence of the human *COQ9* ORF in scHCOQ9 and mcHCOQ9 was confirmed by sequencing (UCLA sequencing core, Los Angeles).

### Disruption of COQ9 in W3031B yeast strain

A PCR product containing the KanMX4 gene was amplified with the genomic DNA isolated from BY4741Δ*coq9* (used as template) with primers that annealed 100 bp upstream and downstream of the *COQ9* ORF. The sequences of the primers utilized were: 5′-TTTGGGCCTACATAAGGTACTTC-3′ and 5′-CGCACAGACCAATAAATCTGCC-3′. The PCR product was then transformed into the yeast W3031B to create W303Δcoq9K. Transformants that grew on YPD + 200 μg/ml G418 (Geneticin) were selected. Proteins were extracted from these transformants as described (Yaffe and Schatz, 1984) and separated by SDS-PAGE with a 10% polyacrylamide gel. Proteins were transferred to an Immobilon-P transfer membrane (Millipore) and analyzed by immunoblotting as described (He et al., 2014). The primary antibody against Coq9 was used at a 1:1000 dilution and the secondary antibody, goat anti-rabbit IgG H&L chain specific peroxidase conjugate (Calbiochem), at a 1:10,000 dilution. The absence of Coq9 polypeptide confirmed that *COQ9* was replaced with KanMX4.

### Lipid extraction and detection of Q_6_-intermediates by HPLC and tandem mass spectrometry

The *de novo* synthesis of Q_6_ and Q_6_-intermediates was tracked in yeast cells labeled with ^13^C_6_-pABA or ^13^C_6_-4HB followed by lipid analysis. Labeling media were prepared with 10 μg/ml ^13^C_6_-pABA or ^13^C_6_-4HB dissolved in ethanol (0.2% final concentration). After 12.5 h of labeling cells were collected (a total of 50 A_600 nm_) as pellets by centrifugation. Q_4_ was added (164 pmol) to each cell pellet to serve as an internal standard. Lipid extracts were analyzed by RP-HPLC-MS/MS (Xie et al., 2012). For liquid chromatography, a phenyl-hexyl column (Luna 5u, 100 × 4.60 mm, 5-μm, Phenomenex) was used. The mobile phase consisted of Solvent A (methanol/isopropanol, 95:5, with 2.5 mM ammonium formate) and Solvent B (isopropanol, 2.5 mM ammonium formate). Solvent B was increased linearly from 0 to 5% with the flow rate increased from 600 to 800 μl/min from 0 to 6 min. The flow rate and mobile phase were changed back to 600 μl/min and 100% Solvent A respectively at 7 min. Multiple reaction monitoring mode (MRM) analysis was performed with the 4000 QTRAP linear MS/MS spectrometer from Applied Biosystems (Foster City, CA). Data were processed with Analyst version 1.4.2 software (Applied Biosystems).

To quantify Q_6_ content, the peak areas of ^12^C-Q_6_ (sum of oxidized and reduced) and ^13^C_6_-Q_6_ (sum of oxidized and reduced) were normalized by the peak areas of Q_4_ (sum of oxidized and reduced); the pmol amounts were then determined from the Q_6_ standard curve. The pmol of ^12^C-Q_6_ and ^13^C_6_-Q_6_ were further normalized by the yeast pellet wet weights. A chemical standard for DMQ_6_ is not available. To quantify this intermediate, the peak areas (sum of oxidized and reduced DMQ_6_) were normalized by the recovery of Q_4_ (sum of oxidized and reduced peaks). Finally, calculated values were further normalized by the yeast pellet wet weights.

### Mitochondria isolation and immunoblot analyses with yeast coq9 temperature-sensitive mutants expressing the human *COQ9* homolog

Yeast cultures were grown to 3–4 A_600 nm_/ml in YPGal medium at different temperatures (W3031B, W303Δ*coq9*:TS19, and W303Δ*coq9*:TS19+mcHCOQ9 were grown at 25 and 37°C for 18.5 h; BY4741ΔCOQ9, W303ΔCOQ7, W303ΔCOQ4, W303ΔCOQ6 were grown at 30°C overnight). Crude mitochondria were isolated from 1 L of culture as described (Padilla-López et al., 2009). Mitochondria were further purified with an OptiPrep discontinuous iodixanol gradient as described (He et al., 2014). The bicinchoninic acid assay was used to measure the total protein concentration in purified mitochondria (ThermoFisher Scientific). Purified mitochondria were solubilized with digitonin as described (He et al., 2014), and 15 μg of mitochondria were separated by SDS-PAGE with 10% polyacrylamide gels. Proteins were transferred to Immobilon-P transfer membranes (Millipore) and immunoblot analyses were performed as described (He et al., 2014). The source and use of primary antibodies are described in Table 2. Goat anti-rabbit IgG H&L chain specific peroxidase conjugate (Calbiochem), 1:10,000 was used as the secondary antibody.

**Table 2:** Description and Source of Antibodies.

**Antibody**	**Working dilution**	**Source**
Atp2	1:4000	Carla M. Koehler^a^
Coq4	1:250	Belogrudov et al., 2001
Coq6	1:250	Gin et al., 2003
Coq7	1:1000	Tran et al., 2006
Coq9	1:1000	Hsieh et al., 2007
Human COQ9	1:1000	Proteintech Group, Inc.

a *Dr. Carla M. Koehler, Department of Chemistry and Biochemistry, UCLA*.

### Co-precipitation of Coq6-CNAP expressing the human COQ9 homolog

Purified mitochondrial proteins (13 mg; 2 mg/ml) were solubilized with 4 mg/ml digitonin as described earlier. The soluble digitonin extract was collected after 100,000 × *g* centrifugation (Optimax TLX). Co-precipitation was then performed on the solubilized mitochondria with Ni-NTA resin (Qiagen) as described in Allan et al. (2015). Briefly, Ni-NTA resin (800 μl bed volume) was equilibrated with two volumes of lysis buffer. Solubilized mitochondria and 8 ml of lysis buffer were added to 800 μl of pre-equilibrated Ni-NTA resin (bed volume) in a 15 ml Falcon tube and rotated for 1.5 h at 4°C. The resin-slurry was applied to a column and the flow-through was collected. The resin was washed twice with Ni-NTA wash buffer (W1 and W2) and eluted twice with Ni-NTA elution buffer (E1 and E2). Aliquots of each fraction were examined for presence of Coq polypeptides: 0.17% of the FT, 0.25% of W1, 0.25% of W2, 1% of E1, 0.5% of E2, and 1.25% of Ni-NTA resin were analyzed by SDS-PAGE followed by immunoblot analysis with antibodies against Coq9, human COQ9 (1:1,000), Coq6, 1:250 and Atp2, 1:4,000. Purified mitochondria (15 μg protein) from CNAP6: *mcHCOQ9* were included as a control.

## Results

### Expression of a human COQ9 homolog rescues the growth of the *Δcoq9k*:TS19 mutant on medium containing a non-fermentable carbon source

**Figure 1:**
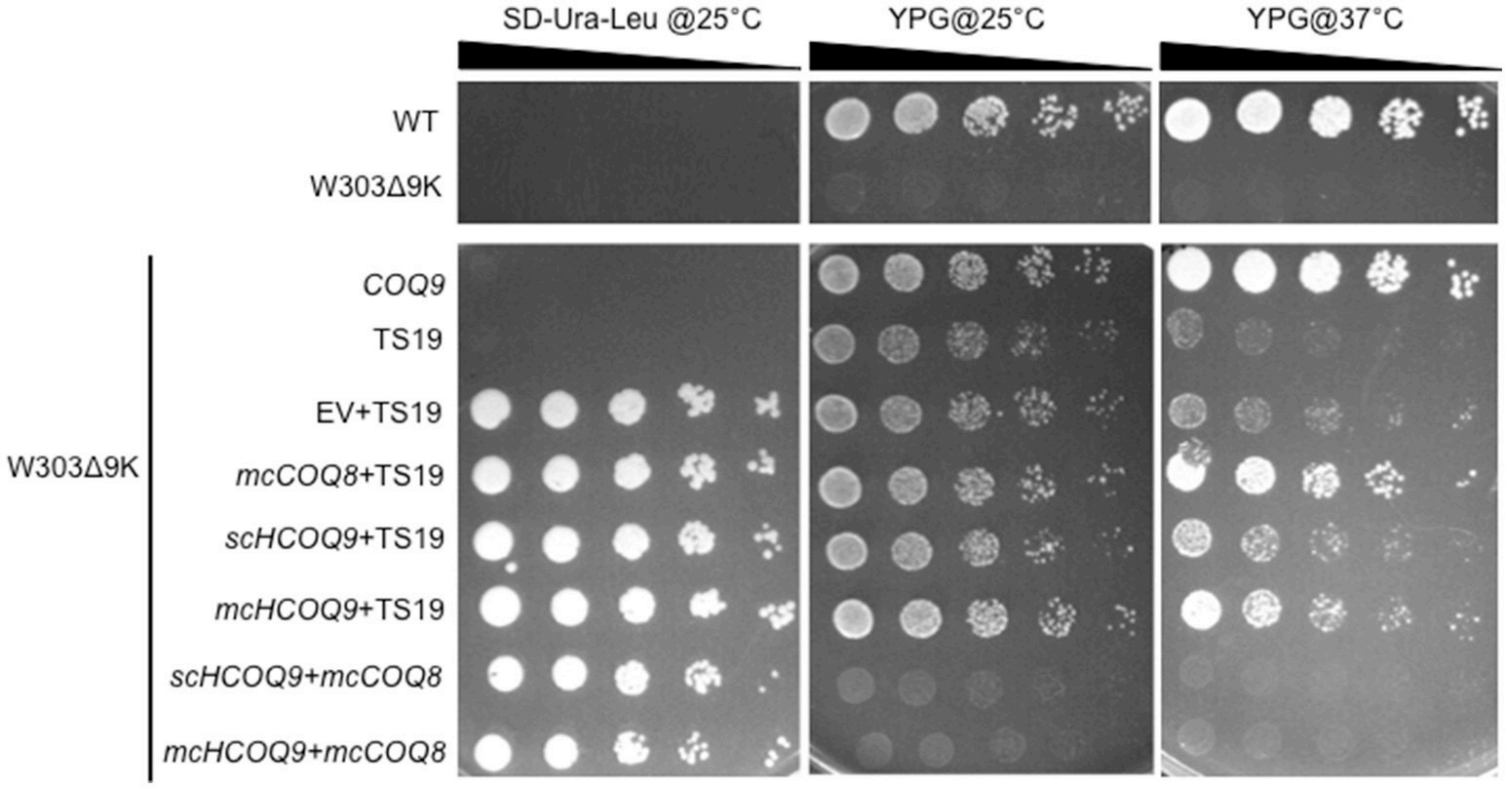
Expression of human *COQ9* or over-expression of *COQ8* rescues the growth of the temperature-sensitive *coq9* mutant on a non-fermentable carbon source. W303Δ9K was transformed with TS19 and one of the following plasmids: empty vector pRS426 (EV), multi-copy yeast *COQ8* (*mcCOQ8*), single-copy human *COQ9* (*scHCOQ9*), and multi-copy human *COQ9* (*mcHCOQ9*). Yeast strains were cultured in SD-Leu-Ura media overnight at 25°C. W3031B (WT), W303Δ9K, W303Δ9K:*COQ9*, and W303Δ9K:TS19 were used as controls and grown in YPG and SD-Leu respectively. Cell cultures were diluted to 0.2 A_600 nm_/ml and 2 μl of 1:5 serial dilutions were spotted onto SD-Ura-Leu or YPG plate media and incubated at either 25°C or 37°C for 3 days.

Expression of human COQ9 has previously failed to rescue yeast *coq9* null mutant growth on non-fermentable carbon sources (Duncan et al., 2009; Hayashi et al., 2014). It seemed likely that the destabilization of other Coq polypeptides in the yeast *coq9* null mutant might account for the inability of human *COQ9* to rescue the mutant. To stabilize other Coq polypeptides in the *coq9* null mutant, we co-expressed multi-copy *COQ8* (*mcCOQ8*) with either single copy (*scHCOQ9*) or multi-copy human *COQ9* (*mcCOQ9*) in a *coq9* null yeast mutant (W303Δ9K) and tested its growth on YPG plate medium, containing glycerol as the sole non-fermentable carbon source. However, expression of *mcCOQ8* alone did not enable human COQ9 to rescue the growth of the *coq9* null mutant (Figure 1).

Next, we turned to the Δ*coq9K:*TS19 mutant, which retains detectable steady-state levels of the yeast Coq9-TS19 polypeptide and other Coq polypeptides, is able to grow on YPG at the permissive temperature (25°C) (He et al., 2015), but shows defective growth on YPG at the non-permissive temperature (37°C; Figure 1). Expression of *mcCOQ8, scHCOQ9*, or *mcHCOQ9* were each able to rescue the growth of the Δ*coq9:*TS19 mutant on YPG at the non-permissive temperature. While expression of yeast *COQ9* yielded the most robust rescue at the non-permissive temperature, expression of *mcCOQ8* and *mchCOQ9* also rescued growth of the *coq9* null mutant, with *schCOQ9* providing noticeably less rescue (Figure 1). At the permissive temperature it is not possible to distinguish the effects of single-copy human *COQ9* or multi-copy *COQ8*. The yeast Δ*coq9K:*TS19 mutant still functions at 25°C, and its growth is similar to wild type. However, there is a slight increase of growth of Δ*coq9K:*TS19 harboring *mcHCOQ9*. Yeast cells were also plated on SD-Ura-Leu to confirm that W303Δ9K was successfully transformed with the two plasmids. The empty vector pRS426, which is the parent vector of *mcCOQ8*, was included as a control (EV). As expected, Δ*coq9K:*TS19 cannot be rescued by the empty vector (Figure 1). Therefore, the rescue effects were specific and depended on the expression of either human *COQ9* or over-expression of yeast *COQ8*.

### In the yeast *Δcoq9k*:TS19 mutant, expression of human COQ9 increased the *De novo* synthesis of Q_6_ from 4HB and over-expression of Coq8 increased the *De novo* synthesis of Q_6_ from pABA

**Figure 2:**
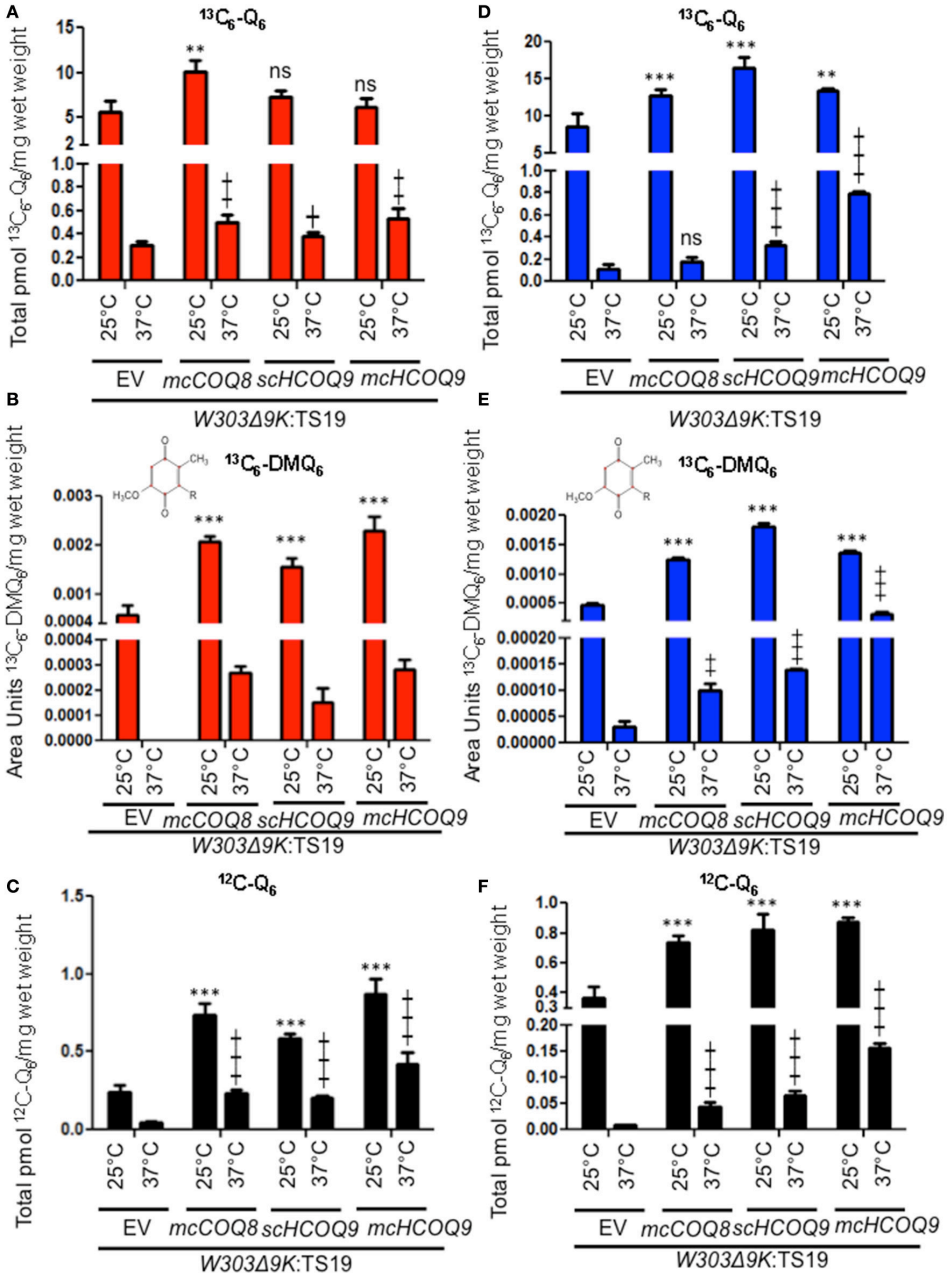
Expression of human *COQ9* or over-expression of *COQ8* increases the content of Q_6_ and DMQ_6_ in W303Δ9K expressing the temperature-sensitive plasmid TS19. W303Δ9K was transformed with TS19 and one of the following plasmids: empty vector pRS426 (EV), multi-copy yeast *COQ8* (*mcCOQ8*), single-copy of human *COQ9* (*scHCOQ9*), and multi-copy of human *COQ9* (*mcHCOQ9*). One colony of each type of yeast transformant was seeded in selective media, SD-Ura-Leu, and grown overnight. The cell culture was diluted to 0.1A_600 nm_/ml in 20 ml of fresh SD-Ura-Leu containing 10 μg/ml ^13^C_6_-pABA **(A–C)** or 10 μg/ml ^13^C_6_-4HB **(D–F)** dissolved in 2 μl ethanol/ml medium and grown at 25 or 37°C for 12.5 h. Final cell density was between 3 and 5 A_600 nm_/ml. Yeast cells (corresponding to a total of 50 A_600 nm_) were collected as pellets, from which lipids were extracted and analyzed by RP-HPLC-MS/MS. Each bar represents a total four measurements from two independent samples each with two injections. Black bars represent the amount of ^12^C-Q_6_, red bars represent ^13^C_6_-Q_6_ and ^13^C_6_-DMQ_6_ labeled by ^13^C_6_-pABA and blue bars represent ^13^C_6_-Q_6_ and ^13^C_6_-DMQ_6_ labeled by ^13^C_6_-4HB. The amounts of the ^12^C- and ^13^C_6_-compounds represent the sum of reduced and oxidized forms. Both Q_6_ and ^13^C_6_-DMQ_6_ levels were higher in W303Δ9K:TS19 harboring human *COQ9* homolog or over-expression of *COQ8* as compared to W303Δ9K:TS19 harboring empty vector as determined by the Student's two-tailed *t*-test. The ^*^ symbols represent samples at 25°C compared to W303Δ9K:TS19+EV at 25°C; ^**^ *p* < 0.01, ^***^p < 0.001. The + symbols represent samples at 37°C compared to W303Δ9K:TS19+EV at 37°C; +*p* < 0.05, ^‡^*p* < 0.01, 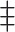
*p* < 0.001. When there is no significant change, ns was used to designate “non-significant.”

Yeast Coq9 is required to remove the nitrogen group from Q_6_ intermediates derived from pABA (He et al., 2015). To assess whether this function is manifested by the expression of human COQ9 in yeast, we determined the *de novo* synthesis of ^13^C_6_-Q_6_ from either ^13^C_6_-pABA or ^13^C_6_-4HB. The yeast Δ*coq9K:*TS19 mutant was transformed with the designated plasmids and levels of Q_6_ and Q_6_ intermediates were determined at permissive and non-permissive temperatures. The presence of *mcCOQ8* significantly increased the amount of ^13^C_6_-Q_6_ synthesized from ^13^C_6_-pABA at the permissive temperature relative to the empty vector control (Figure 2A). At the non-permissive temperature, *mcCOQ8* and both the s*cHCOQ9* and *mcHCOQ9* plasmids increased the amount of ^13^C_6_-Q_6_ synthesized from ^13^C_6_-pABA (Figure 2A). In contrast, *mcCOQ8* and the human *COQ9* homolog increased the amount of ^13^C_6_-Q_6_ synthesized from ^13^C_6_-4HB at the permissive temperature, but only the human *COQ9* homolog increased the amount of ^13^C_6_-Q_6_ synthesized from ^13^C_6_-4HB at the non-permissive temperature (Figure 2D). There is a significant increase of ^13^C_6_-DMQ_6_ in Δ*coq9K:*TS19 with the expression of *mcCOQ8* and both the s*cHCOQ9* and *mcHCOQ9* (Figures 2B,E). Interestingly, *mcHCOQ9* has the most dramatic effect on ^13^C_6_-DMQ_6_ levels when ^13^C_6_-4HB was provided at non-permissive temperature (Figure 2E). These findings suggest that expression of human COQ9 rescues the Δ*coq9K:*TS19 mutant by increasing Q biosynthesis with 4HB as the precursor. The levels of ^12^C-Q_6_ were elevated by *mcCOQ8* and human *COQ9* at both permissive and non-permissive temperatures (Figures 2C,F), perhaps indicating that *mcCOQ8* and human *COQ9* may enhance the utilization of other (unlabeled) ring precursors to increase Q content.

### In the yeast *Δcoq9k*:TS19 mutant, expression of human COQ9 stabilizes yeast Coq polypeptides at permissive temperature

**Figure 3:**
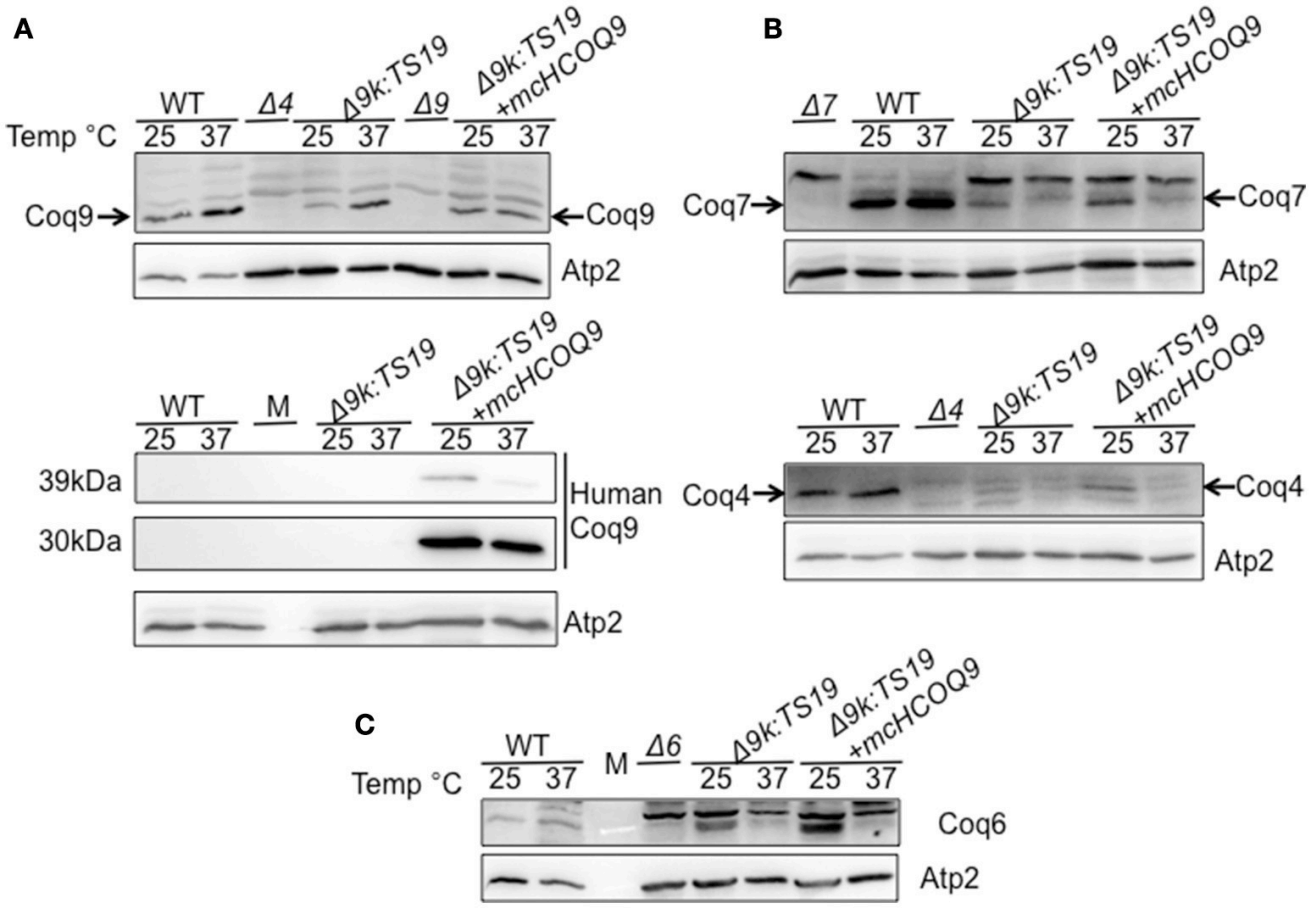
Expression of human COQ9 stabilizes yeast Coq polypeptides in the temperature-sensitive *coq9* mutant at permissive temperature. W303Δ9K harboring the temperature-sensitive plasmid TS19 (Δ9K:TS19) were transformed with multi-copy human *COQ9* (Δ9K:TS19+*mcHCOQ9*). Yeast strains W3031B (WT), Δ9K:TS19, and Δ9K:TS19+*mcHCOQ9* were grown for 18.5 h at either 25 or 37°C. Mitochondria were then purified from these yeast cultures. Mitochondria were also isolated from the null control strains BY4741ΔCOQ9 (Δ9), W303ΔCOQ4 (Δ4) **(A)**, W303ΔCOQ7 (Δ7) **(B)**, and W303ΔCOQ6 (Δ6) **(C)** after yeast were grown overnight at 30°C. Purified mitochondria (15 μg protein) were separated by SDS-PAGE and analyzed by Western blot. Immunoblots were performed with antibodies against the designated polypeptides: Coq4, Coq6, Coq7, Coq9, human COQ9, and Atp2. M denotes the molecular weight marker.

To investigate the mechanisms responsible for the rescue of the Δ*coq9K:*TS19 mutant by human *COQ9*, we determined whether human COQ9 stabilizes other Coq polypeptide levels. Wild type (WT), Δ*coq9K*:TS19 (Δ9K:TS19) and Δ9K:TS19+ *mcHCOQ9* yeast were cultured in YPGal for 18.5 h at either 25 or 37°C, and mitochondria were isolated. Steady-state levels of Coq4, Coq6, Coq7, Coq9, and human COQ9 were analyzed by immunoblotting (Figure 3). The levels of Atp2 were analyzed as a loading control. At the non-permissive temperature, the expression of Coq9-ts19 resulted in lower steady-state levels of the other yeast Coq polypeptides and human COQ9. In contrast, in wild-type yeast, Coq4, Coq6, Coq7, and Coq9 levels were increased at 37°C (Figure 3). In the Δ*coq9K*:TS19 mutant, Coq9-ts19 levels were increased at 37°C (Figure 3A), while steady-state levels of the Coq4, Coq6, and Coq7 polypeptides were decreased at 37°C (Figure 3). The changes of Coq polypeptides in either wild type or the temperature-sensitive mutant at different temperatures did not result from changes in the corresponding *COQ* RNA levels (He et al., 2015). When human *COQ9* was expressed in Δ*9K*:TS19 (Δ9K:TS19+ *mcHCOQ9*), the steady state levels of Coq4, Coq6, Coq7, and Coq9 were significantly increased at the permissive temperature (Figure 3). The results suggest that at permissive temperature the expression of human *COQ9* stabilizes certain yeast Coq polypeptides. Two distinct polypeptides were detected in the mitochondria of Δ*9K*:TS19+*mcHCOQ9* with the antibody against human COQ9. Based on the mass of the polypeptides, it seems likely that the larger polypeptide corresponds to unprocessed human COQ9 with the mitochondrial leader (39 kDa), and the smaller polypeptide represents processed human COQ9 (30.5 kDa). At non-permissive temperature, steady-state levels of human COQ9 were also decreased (Figure 3A). The observed decrease is specific to human COQ9 and Coq proteins of the CoQ synthome because the steady state levels of Atp2, the beta subunit of the F1 sector of the mitochondrial F_1_F_0_ ATP synthase, did not change at higher temperature. These results suggested that human COQ9 may be associated with the CoQ synthome.

### The human COQ9 polypeptide associates with the yeast Q-biosynthetic complex

**Figure 4:**
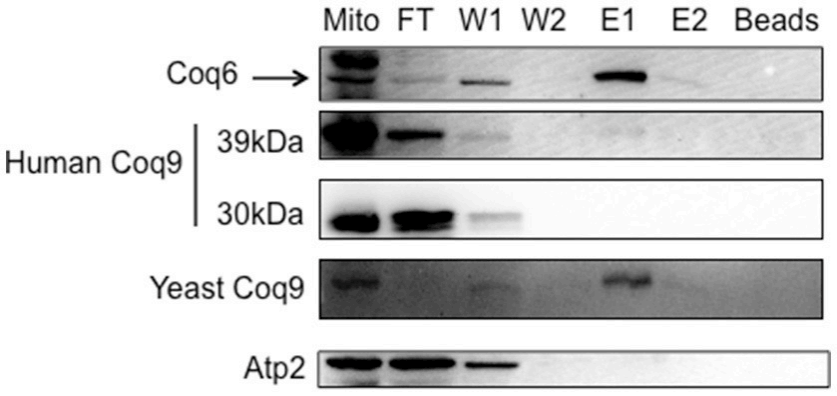
The human Coq9 polypeptide associates with yeast Coq6. Mitochondria were isolated from CNAP6 and CNAP6:*mcHCOQ9*. Purified mitochondria (13 mg) were solubilized and co-precipitation was then performed on the solubilized mitochondria with Ni-NTA resin. Flow-through (FT), wash (W1 and W2), eluate (E1 and E2), and beads from co-precipitation were collected. 0.17% of the FT, 0.25% of W1, 0.25% of W2, 1% of E1, 0.5% of E2, and 1.25% of Ni-NTA resin were analyzed by SDS-PAGE followed by immunoblotting with antibodies against yeast Coq9, Coq6, human COQ9, and Atp2. Purified mitochondria (15 μg) from CNAP6:*mcHCOQ9* were included as control.

To determine whether the human COQ9 polypeptide might interact with yeast components of the CoQ synthome (He et al., 2014), we expressed human *COQ9* in the yeast strain Coq6-CNAP (CNAP6). We chose Coq6-CNAP as the bait protein because Coq9 is known to be important for the Coq6 hydroxylation step (Xie et al., 2012). A consecutive non-denaturing tag containing a His_10_ tag and protein C epitope was integrated at the C-terminus of yeast Coq6, resulting in Coq6-CNAP (Allan et al., 2015). The CNAP6 strain has normal levels of Coq6 and Q_6_ and co-precipitates other Coq polypeptides in the CoQ synthome (Coq4, Coq5, Coq7, Coq8, and Coq9) (Allan et al., 2015). Mitochondria purified from CNAP6:*mcHCOQ9* were solubilized with digitonin and subjected to co-precipitation over Ni-NTA resin. Fractions corresponding to flow through (FT), washes (W1 and W2), eluates (E1 and E2), and beads after elution were analyzed by SDS-PAGE and Western blot. We found that unprocessed human COQ9 and yeast Coq9 co-purified with Coq6-CNAP, (Figure 4). As a negative control, we also blotted with an antibody against Atp2. As expected, Atp2 did not co-purify with Coq6-CNAP (Figure 4). The majority of human COQ9 was detected in the flow through and wash fractions, indicating the interaction between human COQ9 and the Coq6-containing complex is weak or that only a small fraction of the over-expressed human COQ9 interacted with the yeast CoQ synthome (which is not over-expressed).

## Discussion

In this study, the yeast *coq9* temperature-sensitive mutant, Δ*coq9K*:TS19, was rescued by the expression of human COQ9 containing the yeast Coq3 mitochondrial leader sequence. Expression of human COQ9 increased the growth of the Δ*coq9K*:TS19 mutant on medium containing a non-fermentable carbon source (Figure 1), and Q_6_ content (Figure 2). Such rescue was observed at both permissive and non-permissive temperatures. We also found that even though both multi-copy Coq8 and human COQ9 rescued the yeast Δ*coq9K*:TS19 mutant, human COQ9 dramatically increased the level of ^13^C_6_-Q_6_ only when ^13^C_6_-4HB was the precursor (Figure 2C) but not when ^13^C_6_-pABA was the precursor (Figure 2A). In contrast, multi-copy Coq8 increased the level of ^13^C_6_-Q_6_ when ^13^C_6_-pABA was provided (Figure 2A). These findings suggest that human COQ9 increases Q_6_ production by promoting the conversion of 4HB to Q_6_. Our findings are consistent with the observation that yeast can utilize pABA to synthesize Q (Marbois et al., 2010), while human cells are unable to utilize pABA (Xie et al., 2015). A recent study proposed that Coq6, an FAD-dependent monooxygenase, is responsible for the deamination reaction at the C4 position of Q intermediates in yeast (Ozeir et al., 2015). Yeast Coq9 is also required for the removal of nitrogen group of Q intermediates (He et al., 2015), possibly by interacting with the C-terminal region of Coq6, which is important for the C4-deamination (Ozeir et al., 2015). Interestingly, human COQ6 promotes the C4-deamination in yeast in the absence of yeast Coq9 (Ozeir et al., 2015). It is possible that Coq9 is indispensable for the function of Coq6 as a deaminase in yeast, but has an inhibitory effect on the C4-deamination in humans. This could explain the inability of human cells to use pABA as a Q precursor, but this hypothesis will require further investigation.

Human COQ9 failed to rescue yeast *coq9* null mutant, even with the over-expression of yeast Coq8, which is known to stabilize the CoQ-synthome (He et al., 2014; Figure 1). This might be due to the fact that yeast Coq9 is required for the function of yeast Coq6 and Coq7. Yeast *coq6* and *coq9* null mutants over-expressing *COQ8* both accumulate 4-AP when pABA is provided as the ring precursor and yeast *coq7* and *coq9* null mutants over-expressing *COQ8* both accumulate DMQ_6_ when 4-HB is provided (Xie et al., 2012). Yeast Coq6 and Coq7 do not function well without yeast Coq9. The temperature sensitive yeast Coq9-ts19 polypeptide may play a structural or regulatory role that enables human COQ9 to function in yeast. Interestingly, the expression of human COQ9 stabilizes the steady state levels of Coq4, Coq6, Coq7, and yeast Coq9 at permissive temperature. It was shown that supplementation of Q_6_ to yeast mutants stabilizes the CoQ-synthome and its Coq polypeptide subunits (He et al., 2014), hence it is possible that human COQ9 stabilizes yeast Coq proteins by increasing Q_6_ levels. Although we saw that Q_6_ levels of Δ9K:TS19 were significantly increased by human COQ9 at both permissive and non-permissive temperature (Figure 2), human COQ9 stabilizes the steady state levels of yeast Coq polypeptides only at the permissive temperature (Figure 3). This observation may be due to the fact that our LC-MS/MS assay is highly sensitive and small changes in lipid levels are readily detected, while immunoblot assays may not be sufficiently sensitive to reveal small changes in protein levels affected by the expression of human COQ9 at non-permissive temperature.

In order to investigate the mechanism of human COQ9 rescue of the yeast *coq9* mutant, we determined whether human COQ9 is associated with the CoQ-synthome. The structure of human COQ9 was recently identified as a dimer (Lohman et al., 2014). We speculated that human COQ9 might interact with yeast Coq polypeptides. We performed co-precipitation of CNAP-tagged yeast Coq6 and found that a small amount of human COQ9, along with yeast Coq9, co-precipitates with Coq6-CNAP (Figure 4). Therefore, human COQ9 interacts with the yeast CoQ-synthome, reflecting a profound degree of functional conservation. It is unusual that the unprocessed form of hCOQ9 is the sole form recovered together with yeast Coq6-CNAP. The 39kDa form of hCOQ9 persists in isolated mitochondria at the permissive temperature suggesting that mitochondrial processing (i.e., removal of the N-terminal targeting peptide) of the human protein is not as efficient as it is for the yeast protein (assuming that the 39kDa band is the unprocessed form). Perhaps this form may be better suited to interact with Coq6-CNAP, as the 30kDa form of hCOQ9 may be truncated inappropriately or otherwise modified in a way that prohibits this interaction. It does seem apparent that the hCOQ9 will not necessarily interact with the yeast proteins in the exact same way as the yeast Coq9 polypeptide.

We speculate that hCOQ9 may perform a similar enzymatic or structural role as yeast Coq9, however, the presence of the yeast tsCoq9 polypeptide is required to interact and stabilize the other yeast Coq polypeptides. Thus, the yeast tsCoq9 polypeptide may harbor mutations in regions important either for enzymatic functions or protein-protein interactions, which render it less efficient than wild-type Coq9. Expression of hCOQ9 alleviates some of the deficiencies, but at this point we cannot distinguish the exact mechanisms.

In conclusion, we found that human COQ9 interacts with the CoQ-synthome and stabilizes the complex. We speculate that human COQ9 stabilizes the complex by increasing Q_6_ content derived from 4HB; as a result, it rescues the growth of *coq9-ts19* mutant at the non-permissive temperature.

## Author contributions

CH, DB, CA, BM, SR, and CC contributed to the conception and design of the experiments, drafting the manuscript, revising it for intellectual content and approved the final version. CH, DB, CA, and CC contributed to acquisition, analysis, and interpretation of the data.

### Conflict of interest statement

The authors declare that the research was conducted in the absence of any commercial or financial relationships that could be construed as a potential conflict of interest.

## Abbreviations

4-AP: 3-hexaprenyl-4-aminophenol
DDMQ_6_: demethyl-demethoxy Q_6_
DMQ_6_: demethoxy-Q_6_
HAB: 3-hexaprenyl-4-aminobenzoic acid
4HB: 4-hydroxybenzoic acid
HHB: 3-hexaprenyl-4-hydroxybenzoic acid
4-HP: 3-hexaprenyl-4-hydroxyphenol
IDDMQ_6_: imino-demethyl-demethoxy Q_6_
IDMQ_6_: imino-demethoxy-Q_6_
mc*COQ8*: multi-copy *COQ8*
pABA: para-aminobenzoic acid; RP-HPLC-MS/MS; reverse phase-HPLC-MS/MS
Q: Coenzyme Q.
